# High protein diets do not affect anthropometric indexes and cardiometabolic risk factors among children with excess weight: A randomized controlled trial

**DOI:** 10.15171/jcvtr.2018.15

**Published:** 2018-06-27

**Authors:** Vajihe Izadi, Ahmad Esmaillzadeh, Mahin Hashemipour, Pamela J. Surkan, Leila Azadbakht, Roya Kelishadi

**Affiliations:** ^1^Food Security Research Center, Isfahan University of Medical Sciences, Isfahan, Iran; ^2^Department of Community Nutrition, School of Nutrition & Food Science, Isfahan University of Medical Sciences, Isfahan, Iran; ^3^Department of Community Nutrition, School of Nutritional Sciences and Dietetics, Tehran University of Medical Sciences, Tehran, Iran; ^4^Department of Pediatric Endocrinology, Child Growth and Development Research Center, Isfahan University of Medical Sciences, Isfahan, Iran; ^5^Department of International Health, John Hopkins Bloomberg School of Public Health, Baltimore, USA; ^6^Diabetes Research Center, Endocrinology and Metabolism Clinical Sciences Institute, Tehran University of Medical Sciences, Tehran, Iran; ^7^Department of Pediatrics, Child Growth and Development Research Center, Research Institute for Primordial Prevention of Non-Communicable Disease, Isfahan University of Medical Sciences, Isfahan, Iran

**Keywords:** High Protein Diet, Cardiovascular Risk Factors, Children, Obesity, Overweight

## Abstract

***Introduction:*** Limited information exists regarding the effects of high protein (HP) diets on cardiovascular disease (CVD) risk factors among overweight and obese children. Our aim was to determine the effects of an HP diet on anthropometric indexes and CVD risk factors among overweight and obese children.

***Methods:*** In a parallel randomized controlled trial, we recruited 50 overweight and obese children, aged 6-11 years, for a 10 week HP or control diet (protein, carbohydrate, fat: 25%, 45%, 30% in the HP diet vs. 15%, 55%, 30% in the control diet, respectively). Fasting blood glucose (FBG) serum insulin levels, lipid profiles, systolic and diastolic blood pressure (SBP and DBP), and anthropometric measurements were assessed using standard guidelines.

***Results:*** 86% of children completed the trial. Percent changes (PC) for anthropometric and biochemical variables were not significantly different between the two groups. The PC of serum triglyceride (TG) level was significantly decreased in the HP group compared to in the control group (PC: -10.16±4.30% vs.12.11±7.80%; P = 0.01) in the crude model, but not in the adjusted model. For other variables, we did not find any significant differences between the HP group and the controls.

***Conclusion:*** In the present study, we did not find any significant effect of adherence to an HP diet in improving anthropometric measurements or other CVD risk factors among obese and overweight children.

## Introduction

Obesity has emerged as an important public health problem in most countries of the world where its prevalence is rising among children and adolescents at an alarming rate.^1,2^ The prevalence of obesity is two times higher among US children and adolescents compared to during the previous three decades.^3^ In Iran, overweight and obesity are highly prevalent not only in adulthood but also in childhood.^4,5^

Obesity in childhood is a leading cause of many chronic diseases including hypertension, hyperlipidemia, chronic heart disease, stroke, as well as diabetes mellitus and several types of cancers in adults.^6^ Given the important role of obesity in the incidence of many chronic diseases, treatment of obesity in childhood is critical.^2^

Unhealthy diets and a sedentary lifestyle are considered to be the main contributors of overweight and obesity among children.^4^ On the other hand, lifestyle changes, energy restriction with low carbohydrate and low fat diets were among some of the formerly typical childhood treatment options for obesity.^2^ However, several studies revealed that these treatment strategies were not successful and resulted in increases in hunger, and therefore food intake in children.^2,7^ Few studies have examined the effect of a high protein (HP) diet among children. HP diets compared to high carbohydrate and high fat diets may lead to increases in energy expenditure and thermogenesis.^8,9^ Additionally, consumption of HP diets may provide greater satiety and decreased energy intake compared to other macronutrients.^10^ Reduced carbohydrate intake or/and moderate reduction in the diet’s glycemic index may be some of the more beneficial effects of HP diets.^11^ HP diets can maintain normal growth and muscle building ^12^. Given that type of diet plays an important role in childhood’s growth and also that a low calorie diet can have adverse effects on child growth^2,12^; it appears that HP diets are preferable for overweight and obese children.

Since the 1960s, HP diets emphasizing carbohydrate restriction have been popular diet therapies.^13^ HP diets have a beneficial impact on plasma lipid profile, insulin sensitivity and anthropometric measurements among adults.^14^ Results from a recent meta-analysis regarding the effects of a HP weight loss diet on cardiovascular risk factors reinforce this claim.^15^ In contrast, different kinds of proteins have diverse effects on health.^16^ Consumption of animal protein may increase risk of hypertension and urinary calcium excretion.^13,16^

To the best of our knowledge, few studies have been conducted regarding the effects of HP diets on anthropometric measurements in childhood^2,7,10^ and two of these studies did not examined cardio-metabolic risk factors. Furthermore, in most studies, animal proteins were mentioned as protein sources, though these sources of protein may also cause several health problems.^13,16^ Therefore, we investigated the effects of a HP diet composed of 50% plant and 50% animal protein sources on several cardiometabolic risk factors among overweight and obese children.

## Materials and Methods

### Subjects

A total of 50 overweight and obese children ranging from 6 to 11 years old who were not pubertal (parents were asked about children’s the stage of puberty) were recruited and referred to two pediatric clinics in Isfahan, Iran from April to August 2012. Participants were unaware of the study before they were referred to these clinics. The study sample size was calculated based on the equation: n = 2 ([Z_1- α/2_+Z_1-β_]^2^. S^2^)/d^2^ where α (type 1 error) was 0.05, β (type 2 error) was 0.2, S (the variance of LDL) was 2.6 according to one of the national papers and d (the difference in mean of LDL) was 2.5.^20^ We considered LDL our principal outcome variable. Therefore, according to the formula, 17 subjects were needed in each group. After taking into account potential dropout, we sought to enroll 25 children in each group. Children ages 6-11 years who were overweight or obese were included. Overweight was defined as body mass index (BMI) up to the 85th% and obesity was defined as the BMI up to the 95th% based on international reference standards.^1^ Children were included if they did not have known kidney, liver, cardiovascular, allergy, respiratory or thyroid diseases, diabetes, hypercholesterolemia, hypertension and did not have any chronic diseases, congenital or mental diseases and did not take any medication. Participants were excluded if they were not following their prescribed diets, or changed their physical activity levels, did not tolerate the diets, or if any of inclusion criteria were not met. The study protocol and both potential adverse and/or favorable effects of intervention were fully explained to all children by a nutritionist and to one or both of their parents before providing written informed consent.

As part of this randomized parallel design, participants were blocked matched for age, sex and weight by the nutritionist and randomized to one of two groups by based on random numbers. Then a simple random sampling method was used to allocate subjects into two groups (random allocation). Because his was a dietary intervention, patients were not blinded to the kind of dietary intake they received. We could only blind the laboratory staff. Subjects had low physical activity levels at baseline and were asked to continue their usual activity during the trial.

### Diet

Participants were randomly assigned to one of two isocaloric dietary interventions. We used a 100 kcal daily reduction (reduction in calculated daily energy intake based on baseline weight) for overweight and a 200 kcal daily reduction for obese participants for 10 weeks. This was based on two kinds of diets: (1) a HP diet (n = 25); 25% of energy from protein, 45% of energy from carbohydrate and 30% of energy from fat and (2) a control diet (n = 25); 15% of energy from protein, 55% of energy from carbohydrate and 30% of energy from fat. The total amount of protein was divided between animal and plant sources in a 1:1 ratio. Also, animal sources were derived half from meats (e.g. red meat, fish, poultry, egg and other meat products) and half from dairy products (including milk and yogurt). The daily diet menu was selected taking into account a variety of food groups in order to supply all the necessary micronutrients in children.

To facilitate dietary compliance, a qualified dietitian provided dietary advice and meal planning (over 14 days for each participant). Daily meal planning included three main meals, three snacks and one substitution food list. To evaluate whether participants followed the prescribed diets, a dietitian assessed the completion of one food record every two weeks. We also asked participants to complete one baseline food record and calculated energy, based on macro- and micro-nutrients. Analysis of food records was done with Nutritionist IV. Participants were asked to report on the status of their prescribed diet (self-reported and reported by a parent) and on any potential problems or questions. To assess adherence to the diets prescribed we used the Maroni formula along with urinary urea nitrogen (UUN).^17^

### Anthropometric and blood pressure measurements

Subjects were weighed every 14 days using analog scale to the nearest 100 g while wearing light clothing, no shoes and following fasting overnight. Height was measured to the nearest 0.5 cm with non-stretchable tape at week zero and subsequently every 2 weeks. For this procedure, subjects remained barefoot in the standing position with their shoulders in a standard position. BMI was calculated by weight (kg)/height (m^2^).^3^ At baseline and every two weeks thereafter, waist circumference was measured with non-stretchable tape at the narrowest point over light clothing without any pressure on the body surface. Hip circumference was measured to the nearest 0.5 cm at the maximum width of the hip without any pressure on the body surface.

Systolic and diastolic blood pressure (SBP & DBP) were measured in duplicate after at least 5 minutes of rest in a calm sitting position, with appropriate cuff-sizes according to arm size. Trained technicians measured SBP, which was defined as the clear of the first sound (first Korotkoff phase) and DBP was defined as the disappearance of sound (fifth Korotkoff phase). The average of the two BP measurements was recorded and included in the analysis.

Data on physical activity were asked by using the physical activity record at the beginning and the end of study and activity was classified using light, medium and heavy levels in International Physical Activity Questionnaire (IPAQ). Then metabolic equation hours per weeks (MET.h/day) were calculated for each subject.^18^

### Biochemical measurements

Total 24-hour urine was collected (except from the first morning urine) at baseline and the end of the study using the Maroni formula. According to a standard protocol, fasting blood samples (10 mL) were collected at baseline and the end of the study while children were in the resting position. Samples were centrifuged within 30-45 minutes of collection for 10 minutes at 500×g and at 4^°^C. Samples were analyzed using an auto-analyzer (Selectra 2; Vital Scientific, Spankeren, Netherlands). HDL cholesterol, LDL-c, fasting glucose and total cholesterol were measured using an enzymatic kit (Pars Azmmoun, Tehran, Iran). Triglyceride was measured with glutathione oxidase. Protein intake UUN was determined based on assessment of UUN using the Maroni formula: (protein intake (gr/kg/d) = UUN+ 0.031×weight (kg) ×6.25).^17^ We calculated the homeostatic model for insulin resistance assessment (HOMA-IR) and quantitative insulin sensitivity check index (QUICKI) based on the following formulas^19^:

HOMA-IR = ([glucose (mmol/L)] [insulin (mU/L)])/22.5

QUICKI = 1/ (log [insulin] +log [glucose])

### Statistical analysis

The normality of the distribution of each variable was checked with the Kolmogorov-Smirnov test. All variables had a normal distribution. Baseline and end values of cardiovascular risk factors including weight, waist circumference, LDL-C, HDL-C, total cholesterol, fasting blood glucose (FBG), triglyceride (TG), Apo protein A and B100, fasting blood insulin, Apo A/Apo B, TC/HDL, TG/HDL, non HDL cholesterol, HOMA-IR index, QUICKI index, SBP, DBP and SBP to DBP ratio in the HP diet and control diet groups were compared using independent sample *t* tests. P values of within-group analysis of the two groups were assessed with paired *t* tests. End point and baseline of treatment values were used to calculate the percent change of each variable. Percent change in cardiovascular risk factors in the HP diet and control diet groups were compared using independent *t* tests. Analysis of variance (ANOVA) was used to adjust for the effects of baseline waist circumference on cardiovascular disease (CVD) risk. The P values of possible interactions for time, type of diet and time × group were also reported. We used SPSS software (version 16.0; SPSS Inc., Chicago IL) for the statistical analyses. *P*<0.05 was considered statistically significant.

## Results

**Figure 1:**
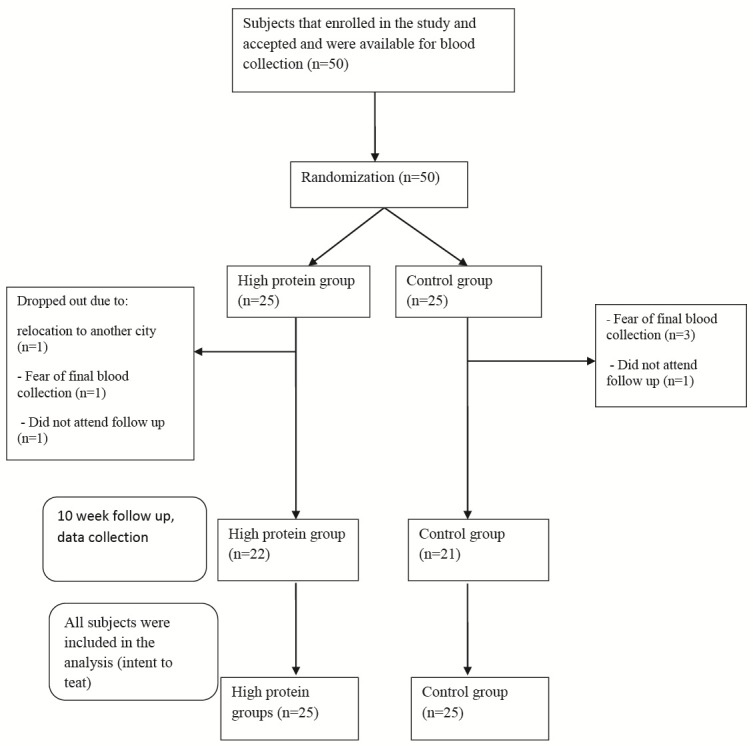
Participant flow diagram of the study.

Of 50 participants (31 girls with BMI = 21.53±1.58 kg/m^2^ and age 8.42±1.28 years and 19 boys with BMI = 23.00±1.84 kg/m^2^ and age 9.33±1.62 years) who enrolled in this study, 43 subjects completed the study and 7 subjects dropped out due to: (1) fear of final laboratory tests (n = 4), (2) not attending the clinic at follow up (n = 2) or (3) relocation to another city (n = 1) (Figure 1). However, we used intention to treat analysis and included all the subjects in the analysis. The mean ± standard errors (SEs) for age and BMI of participants were 8.76±0.20 years and 22.09±0.27 kg/m^2^, respectively. The numbers of girls and boys enrolled in the study were 31 and 19; respectively, of which 17 (6 boys and 12 girls) and 33 (14 boys and 18 girls) were overweight and obese, respectively. There were no significant differences between the two groups for age, sex or weight (*P* = 0.1, 0.1, 0.07, respectively) (data not shown). Children did not report any specific unfavorable effects. UUN and the results of Maroni formula were substantially elevated in the HP group compared to the control group (*P* = 0.0001) (Table 1). This indicates compliance of the HP group to the prescribed diet.

**Table 1:** Mean ± SE of urinary variables of overweight and obese children after receiving a high protein diet or control diet

**Variables**	**Control group** ^ 1 ^ ** (n=25)**	**High protein group** ^ 2 ^ ** (n=25)**	***P***
Urinary urea nitrogen (g/kg/d)	7.78±1.55	12.20±2.40	0.0001
Urinary protein 24 h (mg/dL)	22.20±4.40	32.50±6.50	0.08
Protein intake resulted from Maroni^3^ (g/kg/d)	3.20±1.64	6.70±2.40	0.0001

^1^The control diet was defined as a diet with protein, carbohydrate, fat: 15%, 55%, 30% , respectively. ^2^The high protein diet was defined as a diet with protein, carbohydrate, fat: 25%, 45%, 30%, respectively. ^3^The Maroni formula was calculated based on protein intake (g/kg/d) = UUN+ 0.031×weight (kg)×6.25)

The mean ± SE of participants’ dietary energy and nutrient intakes are reported in Table 2. Both diets were well tolerated among all subjects and no adverse effects were reported during the trial period. Findings from dietary records showed that mean protein and carbohydrate intakes were significantly different between the two groups. The mean energy intake between the HP and control groups were 1774.40±77.10 and 1774.29±91.92 kcal, respectively (*P* = 0.8). Dietary B_12_ in the HP group was significantly higher than in the control group (*P* = 0.03). Other water and fat soluble vitamins were not substantially different between the two dietary groups. Physical activity during the study was not significantly different between the two groups (HP: 34.60±1.51 and control: 32.58±1.72 MET.h [metabolic equivalent of task]/day, *P* = 0.6).

**Table 2:** Energy and nutrients intakes of children in the high protein diet and control diet groups during the study

	**Control group^1^ (n=25)**	**High protein group^2^ (n=25)**	***P***
Energy (kcal/d)	1774.40±91.92	1774.20±77.10	0.80
Protein (g/d)	64.59±3.93	95.60±4.67	0.0001
Carbohydrate (g/d)	234.80±10.83	173.40±8.17	0.0001
Fat (g/d)	82.80±5.06	86.52±3.75	0.55
B12 (mcg/d)	2.89±0.366	4.31±0.53	0.03
Vitamin D (mg/d)	1.58±0.42	1.86±0.36	0.6
Vitamin C (mg/d)	73.70±7.09	138.30±38.40	0.19
Vitamin A (mcg/d)	1117.20±275.70	1776.40±1020.95	0.50
Cholesterol (g/d)	280.90±126.40	268.20±123.54	0.90
SFA (g/d)	262.10±21.20	275.90±24.13	0.67
MUFA (g/d)	16.05±2.58	18.71±3.67	0.55
PUFA (g/d)	12.10±1.36	18.26±7.67	0.43

SFA: saturated fatty acids; MUFA: mono un-saturated fatty acids; PUFA: poly unsaturated fatty acids ^1^ The control diet was defined as a diet with protein, carbohydrate, fat: 15%, 55%, 30%, respectively. ^2^ The high protein diet was defined as a diet with protein, carbohydrate, fat: 25%, 45%, 30%, respectively.

The baseline and end values of anthropometric and biochemical variables are presented in Table 3. Only baseline waist circumference differed substantially between the two groups (*P* = 0.03). The end point of weight, waist, BMI, hip circumference, waist to hip circumference, total cholesterol, LDL, HDL, DBP and SBP, Apo A, Apo B_100_ and insulin were not significantly different between the two groups. However, FBS was significantly decreased in the HP group (*P* = 0.01). Serum TG level was marginally significantly decreased in the HP group (*P* = 0.06). Baseline height was the same between the two groups. The height end values were 135.60±1.86 and 139.50±1.80 cm in the control and HP groups, respectively (*P* = 0.09).

**Table 3:** Baseline and end values^1^ of cardiovascular risk factors in the high protein diet and control diet groups

**Variables**	**Control diet group** ^2^ **(n=25)**	**High protein diet group** ^3^ ** (n=25)**	***P*** **value**
Weight (kg)			
Baseline	38.82±1.59	42.92±1.55	0.07
End	38.90±1.59	42.20±1.52	0.10
*P*	0.60	0.02	
Waist (cm)			
Baseline	76.34±1.06	80.06±1.27	0.03
End	73.80±1.54	75.36±1.04	0.30
*P*	0.0001	0.0001	
BMI (kg/m^2^)			
Baseline	21.69±0.39	22.48±0.38	0.10
End	21.07±0.44	21.50±0.39	0.10
*P*	0.003	0.0001	
Hip circumference (cm)			
Baseline	83.52±1.31	86.80±1.22	0.07
End	82.42±1.39	83.76±1.09	0.40
*P*	0.007	0.0001	
Waist to hip circumference			
Baseline	0.91±0.007	0.92±0.009	0.50
End	0.89±0.007	0.90±0.008	0.70
*P*	0.01	0.001	
Triglyceride (mg/dL)			
Baseline	122.40±19.43	106.44±7.80	0.40
End	129.80±19.63	91.80±5.27	0.06
*P*	0.3	0.01	
Fasting blood glucose (mg/dL)			
Baseline	91.56±1.26	89.52±0.93	0.20
End	92.64±1.05	89.20±.0.90	0.01
*P*	0.70	0.20	
Total cholesterol (mg/dL)			
Baseline	156.52±6.15	169.32±4.81	0.10
End	162.24±7.44	167.44±4.38	0.50
*P*	0.20	0.70	
LDL cholesterol (mg/dL)			
Baseline	89.40±4.65	94.12±4.44	0.40
End	95.48±4.86	91.92±3.24	0.50
*P*	0.10	0.50	
HDL cholesterol (mg/dL)			
Baseline	43.92±1.72	47.32±1.35	0.12
End	44.80±2.56	44.80±1.51	0.10
*P*	0.70	0.03	
Systolic blood pressure (mm Hg)			
Baseline	107.04±2.54	111.56±2.19	0.10
End	102.96±2.91	104.98±1.91	0.50
*P*	0.10	0.02	
Diastolic blood pressure (mm Hg)			
Baseline	65.34±2.42	66.26±1.38	
End	66.00±2.61	64.98±1.60	
*P*	0.80	0.50	
Systolic to diastolic blood pressure			
Baseline	1.65±0.03	1.69±0.03	0.50
End	1.58±0.04	1.62±0.03	0.40
*P*	0.10	0.20	
Apo A (mg/dL)			
Baseline	121.20±4.89	129.68±2.80	0.10
End	123.40±5.32	168.08±40.30	0.20
*P*	0.60	0.30	
Apo B100 (mg/dL)			
Baseline	84.00±4.01	90.28±2.70	0.20
End	70.64±4.50	76.04±3.06	0.30
P	0.002	0.0001	
Apo A to Apo B100			
Baseline	1.49±0.06	1.47±0.05	0.70
End	1.87±0.11	2.35±0.63	0.40
*P*	0.001	0.10	
Insulin (mg/dL)			
Baseline	11.11±1.15	13.07±1.07	0.20
End	10.81±0.95	12.24±0.79	0.20
*P*	0.80	0.30	
TC/HDL			
Baseline	3.59±0.10	3.16±0.97	0.93
End	3.69±0.10	3.77±0.70	0.52
*P*	0.0001	0.0001	
TG/HDL			
Baseline	2.80±0.41	2.28±0.18	0.25
End	3.03±0.44	2.09±0.13	0.04
*P*	0.0001	0.0001	
Non-HDL cholesterol (mg/dL)			
Baseline	126.60±5.11	122.00±4.28	0.16
End	117.44±6.34	122.64±3.35	0.47
*P*	0.54	0.74	
HOMA-IR^4^			
Baseline	6.60±0.72	7.51±0.60	0.36
End	6.39±0.55	7.06±0.48	
*P*	0.56	0.71	
QUICKI^4^			
Baseline	0.43±0.00	0.42±0.00	
End	0.43±0.00	0.42±0.00	0.10
*P*	0.80	0.60	

^1^ Values are mean±SEM ^2^ The control diet was defined as a diet with protein, carbohydrate, fat: 15%, 55%, 30% , respectively. ^3^ The high protein diet was defined as a diet with protein, carbohydrate, fat: 25%, 45%, 30%, respectively. ^4^ These indices were calculated based on present formulas: HOMA-IR= ([glucose (mmol/L)] [insulin (mU/L)])/22.5 and QUICKI= 1/(log[insulin]+log[glucose]

Table 4 indicates the percent changes (PC) of anthropometric and biochemical variables. The percent change of serum TG level was significantly decreased in the HP group compared to the control group (PC: -10.16±4.30% vs. 12.11±7.80; P = 0.01) in the crude model, but we did not find any significant differences between the two groups after adjustment for baseline waist circumference. Only in the crude model, a more significant reduction was observed in percent change of hip circumference in the HP group compared to the control group (*P* = 0.01). Reduction in waist circumference was not statistically significant, when comparing the in HP diet group with the control group (PC: -5.70±0.88% vs. -3.50±0.72%; *P* = 0.06).

**Table 4:** Percent changes^1^ of cardiovascular risk factors in high protein and control diet groups

**Variables**	**Control groups** ^2^ **(n=25)**	**High protein groups** ^3^ ** (n=25)**	**Model 1** ^4^ ***P***_values_	**Model 2** ^5^ ***P***_values_	***P***_time_	***P***_group_	***P***_time×group_
Weigh	-0.23±0.60	-1.63±0.73	0.14	-	0.13	0.10	0.06
Waist circumference	-3.55±0.72	-5.70±0.88	0.06	-	0.0001	0.09	0.02
Hip circumference	-1.57±0.52	-3.4±0.48	0.01	0.33	0.0001	0.10	0.005
BMI	-2.87±0.85	-4.3±1.01	0.20	0.43	0.0001	0.20	0.20
Waist to hip	-1.90±0.72	-2.36±0.64	0.60	0.29	0.0001	0.60	0.60
TG	12.11±7.80	-10.16±4.30	0.01	0.76	0.40	0.10	0.03
FBS	1.35±0.93	-0.21±1.07	0.20	0.56	0.50	0.04	0.20
Insulin	8.73±9.06	3.16±8.70	0.60	0.91	0.40	0.10	0.70
LDL	9.73±5.44	1.12±4.36	0.20	0.15	0.40	0.90	0.10
HDL	3.49±6.23	-4.88±2.47	0.20	0.77	0.50	0.40	0.20
Total cholesterol	4.29±3.43	0.04±2.90	0.30	0.24	0.50	0.20	0.20
SBP	-3.07±2.50	-5.15±2.26	0.50	0.31	0.008	0.20	0.50
DBP	1.61±3.69	-0.90±2.97	0.50	0.74	0.80	0.90	0.50
SDBP	-2.77±3.69	-3.01±2.46	0.90	0.06	0.06	0.20	0.80
Apo A	3.33±4.05	29.03±29.25	0.30	0.77	0.30	0.30	0.20
Apo B100	-14.60±4.09	-15.30±3.01	0.80	0.75	0.0001	0.20	0.80
Apo A/Apo B100	26.72±6.38	60.84±41.71	0.40	0.84	0.05	0.40	0.40
TC/HDL	3.89±3.71	5.50±2.24	0.71	0.73	0.05	0.69	0.60
TG/HDL	12.24±7.71	-3.38±5.26	0.10	0.83	0.90	0.10	0.08
Non-HDL cholesterol	4.91±4.16	2.60±3.61	0.67	0.35	0.38	0.24	0.50
HOMA-IR	-10.40±9.29	-3.50±9.18	0.59	0.93	0.44	0.28	0.78
QUICKI	-0.21±1.81	-1.09±1.71	0.70	0.20	0.80	0.10	0.60

^1^ Values are Mean±SEM ^2^ The control diet was defined as a diet with protein, carbohydrate, fat: 15%, 55%, 30% , respectively. ^3^ The high protein diet was defined as a diet with protein, carbohydrate, fat: 25%, 45%, 30%, respectively. ^4^ Model 1 is the crude model (without adjustment), ^5^ Model 2 is adjusted for the baseline waist circumference.

We observed a marginally significant decrease in SDBP (systolic to diastolic blood pressure) between the two groups after adjustment for waist circumference (*P* = 0.06). The PC of other variables were not significantly different between the two dietary groups.

## Discussion

The results of the present study showed that a HP diet did not significantly reduce weight and other cardiovascular risk factors except for WC and serum triglyceride levels among overweight and obese children. To our knowledge, the present study is the first to investigate the effects of randomization to a HP diet from both plant and animal sources on cardiovascular risk factors among overweight and obese children.

Consumption of a HP diet did not significantly affect anthropometric measurements compared to consumption of a control comparison diet. This result is consistent with two another studies on children,^7,10^ while another study found effective weight loss occurred following a HP diet among obese children.^2^ The results from a recent meta-analysis of high-protein weight-loss diets on anthropometric measurements showed that HP diets with calorie reduction could significantly improve weight, BMI and waist circumference among obese adults.^15^ However, results for adults and children may be different. In the present study we did not try to achieve high levels of weight loss, because the target group was children. The slight decrease in the caloric intake in the present study was planned based on standard guidelines to help both intervention and control groups achieve benefits during the study. So, it is not logical to compare our results with other studies among adults (given unknown differences between adults and children).

We did not find a statistically significant effect following adherence to a HP diet on fasting blood sugar (FBS), insulin and HOMA-IR in overweight and obese children in within group analyses. This might be related to the association between insulin status and protein intake.

In the present study, adhering to a HP diet for 10 weeks did not change the lipid profile status of overweight and obese children compared to that of the control group. This may be due to approximately normal levels of lipids in children at the outset. In another study, adhering to a HP diet also did not substantially reduce lipid profiles levels compared to the control group among obese children.^2^ The ratios of TC/HDL, TG/HDL, Apo A/Apo B and non- HDL cholesterol (the amount of cholesterol carried by all lipoproteins except HDL that predict risk of CVDs) also did not significantly change in our study. It seems that non-HDL-C is considered a better predictor of non-lipid CVD risk factors among children who are obese. Measurement of this variable is recommended for obese children.^22^ High levels of TG/HDL are strongly correlated with insulin resistance in children, as the TG/HDL>3 is extremely specific for insulin resistance.^23^ In addition, TC/HDL strongly predicted the risk of coronary heart diseases independent of age, body weight and history of chronic diseases.^24^ In our study, SDP and DBP did not differ substantially between the intervention and the control group. Improvement in the HDL-C and SBP in intra group analysis after HP diet might be due to significantly weight loss in HP diet group.

We did not find any significant effects of adhering to a HP diet on anthropometric indicators and/or cardiovascular risk factors. Reduction in triglyceride levels can be due to adherence to a low carbohydrate diet.^13^ Also, reducing glucose levels after a meal by decreasing intake of carbohydrate and increasing protein in the diet can reduce future risk of CVD. Calcium in dairy products also can improve high blood pressure.^25^

Based on published research, proteins have more satiating characteristics than fats and carbohydrates. Increased consumption of protein from 15% to 30% produced a decrease in dietary intake. Protein is the most satiating macronutrient. Thus, it may facilitate weight loss over the longer term 26, which can play an important role in the improvement of body weight among obese children. High carbohydrate intake can decrease HDL levels compared to a HP diet.^27^ We previously showed that a HP weight loss diet decreased body weight and waist circumference of obese women.^28^ These results may occur due to caloric restriction in adult women. A HP diet also may improve the lipid profile, independent of weight loss.^25^ Increased protein intake in obese hyper-insulinemic subjects significantly decreased the TG level (29%) after 12 weeks.^13^

Several possible factors may account for the lack of significant benefits observed in our study. First, because the children were in a critical period for their growth, we did not decrease children’s caloric intake at a high rate. Also, we prescribed dairy products in combination with meats to achieve a HP intake. In contrast, other studies have examined the effects of dairy or meats separately on CVD risk factors. The difference between protein and carbohydrate intake between the HP group and the control group was 10% of total energy intake. A higher difference in protein intake between the intervention and control groups may be necessary to observe an impact. It was difficult to achieve decreases in FBG, lipid profile and blood pressure in children with approximately normal initial levels, particularly in the short term (only 10 weeks), as well as improvements in body weight and BMI.

The present study has some strengths and limitations. This is the first study to assess adherence to a HP diet based on equal amounts of plant and animal proteins and including low fat dairy products. Because participants were both girls and boys, these results are applicable to obese and overweight children generally. Measuring compliance to a prescribed diet is difficult among children and dropout in studies with children is typically high. However, in the present study we tried to minimize dropouts by regular fortnightly follow up sessions, therefore increasing the accuracy of the study. Moreover, we used the Maroni formula to measure compliance with the HP diet as well as dietary records. The results indicated very good compliance of children in the study. The short duration of the present study is one of the important limitations. In the future additional studies with more participants followed over a longer period of time are needed to fully understand the effects of HP diets on cardiovascular risk factors in overweight and obese children.

In summary, in our study, we did not find any meaningful impact of adherence to a HP diet on the improvement of anthropometric indicators or cardiovascular risk factors in obese and overweight children except for serum TG level.

## Ethical approval

This study was approved by the Isfahan University of Medical Sciences, Isfahan, Iran (research project number 190169). This trial was registered at Clinical trial.gov NCT01886482 and also was registered at the Iran Clinical Trials Center, ID number IRCT201307232839N6.

## Competing interests

All authors declare no competing financial interests exist.

## Acknowledgments

The Isfahan University of Medical Sciences supported this study. The authors express their gratitude to the participants. We are also grateful to the Milad laboratory of Isfahan.
